# Do Bird Friendly® Coffee Criteria Benefit Mammals? Assessment of Mammal Diversity in Chiapas, Mexico

**DOI:** 10.1371/journal.pone.0165662

**Published:** 2016-11-23

**Authors:** S. Amanda Caudill, Robert A. Rice

**Affiliations:** Smithsonian Conservation Biology Institute, National Zoological Park, MRC 5503, Washington, D.C., 20013, United States of America; University of South Carolina, UNITED STATES

## Abstract

Biodiversity-friendly coffee certifications offer a viable way to protect wildlife habitat while providing a financial incentive to farmers. Most studies related to these certifications focus on avian habitat requirements and it is not known whether these standards also apply to other wildlife, such as mammals, that inhabit the coffee landscapes. We assessed the non-volant mammalian fauna and their associated habitat requirements in 23 sites representing forest, Bird Friendly® shade, conventional shade, and sun coffee habitats. We used Sherman trap-grids to measure small mammal abundance and richness, while camera traps were set for medium-sized and large mammals. We detected 17 species of mammals, representing 11 families. This preliminary study indicates that coffee farms in this region provide an important refuge for mammalian wildlife. Mammal species density ranked significantly higher in Bird Friendly® coffee sites than other coffee habitats, although there was no significant difference for species richness (using Chao2 estimator) among the habitat types. No significant difference was found in small mammal abundance among the habitat types. We found a higher species density of medium and large mammals in sites with larger, more mature shade trees associated with, but not required by Bird Friendly® certification standards. However, lower strata vegetation (5 cm to 1 m tall), the only vegetation parameter found to increase abundance and density for small mammals, is not specified in the Bird Friendly® standards. Our findings suggest that although the standards devised for avian habitat do benefit mammals, further study is needed on the requirements specific for mammals that could be included to enhance the coffee habitat for mammals that inhabit these coffee landscapes.

## Introduction

Given the prevalence and predicted expansion of agricultural lands, forest reserves alone cannot be relied upon for conservation of biodiversity. Other conservation strategies must be employed in conjunction with preserving natural habitats if we are to protect and procure the survivability of our world’s biodiversity. Coffee is grown in tropical regions of the world that often overlap with biodiversity hotspots. Managing coffee farms in a way that promotes and protects wildlife habitat is one such conservation strategy that can provide a refuge for biodiversity. The importance of enhancing biodiversity within tropic agricultural landscapes cannot be overstated.

Mexico is considered a biodiversity hotspot [1] and ranks as the world’s fifth most biodiverse country [2]. It also ranked sixth in the world for the highest deforestation rates from 1990 to 2000 [3], although the deforestation rate has declined from 0.52% in 2000 to 0.24% in 2010 [4]. Chiapas is one of the three states where most of this deforestation has occurred [5]. Connecting the remaining forested areas with high quality matrix within this fragmented landscape [6] is imperative for the survival of the wildlife in this region.

Shade coffee is increasingly recognized as an important reservoir for biodiversity and a high quality habitat for wildlife in the agriculture-forest landscape matrix. Research shows that high floristic complexity and vegetative structure within shade coffee farms provides a refuge for birds, insects, and bat communities [7–9]. Most of the research for biodiversity in agroforestry has focused on birds and insects, and while mammal studies in this field are few, their numbers are increasing. The handful of non-volant mammal studies indicates that shade coffee may provide a refuge for mammals as well [10–13].

All taxonomic groups do not respond the same to shade cover gradients within agroforestry landscapes. Perfecto et al. [14] found no correlation between species richness of birds, butterflies, and ants across a coffee intensification gradient in a tri-taxa comparison in Chiapas. Similarly, in another study in Mexico, Pineda et al. [15] found different responses for frogs, bats, and dung beetles in comparing diversity between cloud forest and shade coffee plantations. These studies indicate that different taxonomic groups cannot serve as surrogates for one another, as habitats are used differently among taxonomic groups, guilds, and species, each with their own specific habitat requirements. Birds and other volant groups, for example, may move easily between habitat patches, whereas non-volant mammals may be more immediately effected by patch size, shape, degree of isolation and connectivity [16–20]. These studies highlight the importance of researching various taxonomic groups that inhabit these landscapes.

Shade coffee programs provide a viable market-based approach to enhance wildlife habitat and provide financial incentives for farmers to maintain or re-establish shaded systems within their coffee farms [21–24]. Coffee certifications created to promote biodiversity have developed standards and management guidelines to enhance wildlife habitat by requiring high levels of shade cover and native vegetation complexity [22–23]. The most robust shade certification standards were developed for the Smithsonian Bird Friendly® (BF) coffee program from migratory bird studies and as such are based on requirements for avian habitat. It is not known whether these same standards apply to enhancing habitat within coffee farms for non-volant mammals.

For a farm to be certified as BF, it must meet a stringent set of management standards. The prerequisite is that the farm be certified organic. In terms of vegetation standards, BF certified farms must contain a minimum of 11 different shade tree species, all of which must represent at least 1% or more of all individuals, with the main (“backbone”) species accounting for no more than 60% of all individuals. The principal canopy layer must be at least 12 meters in height. Bird Friendly farms must maintain a minimum of 40% shade tree cover and epiphytic plants are encouraged. Additionally, three distinct shade strata should comprise the canopy of a BF farm.

Although studies have been conducted on the benefits of shade coffee for biodiversity, little research has been done on the effectiveness of the shade certifications to enhance wildlife habitats [22]. Only five studies, to our knowledge, have been published in the recent past examining wildlife biodiversity and shade certification status (all based on Bird Friendly criteria), all in the Soconusco region on Chiapas [5,7,22,25,26]. Collectively, these studies assessed ant, bird, and butterfly diversity within coffee farms of varying shade cover and management intensity. The results revealed that for ants and birds, more forest species were found in BF farms than in non-BF farms. For butterflies, the species richness was higher in BF farms, but there was no difference in the number of forest species between the two farm types. Generally, ant, bird, and butterfly species richness increased with a decrease in management intensity on coffee farms [5,22]; although in the Perfecto et al. [7] study there were conflicting results between study sites for bird richness.

The two studies published on non-volant mammals in the coffee-growing area of Chiapas focused on comparing the mammal diversity in shade coffee and adjacent forest. Cruz-Lara et al. [11] found that mammalian species richness and diversity were highly similar between shade coffee sites and tropical rainforest. In contrast, Sáenz and Horváth [13], who compared mammal diversity in a cloud forest reserve and adjacent shade coffee sites, found a low degree of similarity in species composition between these two habitats for small non-volant mammals, although the mammal diversity was not significantly different. Both studies looked at discrete habitat types and neither compared gradients of shade cover or shade certification standards within the coffee farms.

Even though the number of mammal studies within agroforestry is increasing, many gaps remain in our knowledge of how mammals respond to varying degrees of shade cover and management intensity within coffee farms and how we can best enhance this potential refuge for mammals in these fragmented landscapes. This is the first study, to our knowledge, that seeks to determine if the same standards for shade certifications developed for avian fauna also apply to mammalian fauna. In addition to further understanding the ecology of mammal communities in coffee landscapes, our study was devised to determine if the coffee farms that meet the BF criteria differ in the abundance and richness of mammals compared to unshaded farms and conventional shaded farms that do not meet the stringent BF standards.

In this initial study of mammals in coffee agroforestry, we investigated the non-volant mammalian fauna and vegetation characteristics within 5 forest sites and 18 coffee sites with varying levels of shade cover in the Soconusco region of Chiapas. The objectives of our study are as follows: (1) Assess the mammal diversity within coffee farms of varying degrees of shade cover and vegetation complexity; (2) Quantify the habitat parameters associated with observed diversity and abundance of mammals; (3) Evaluate the current Smithsonian Bird Friendly® standards for enhancing mammal habitat; and (4) Summarize the suggested management practices to enhance mammal habitat within coffee farms.

## Materials and Methods

Our study took place in the mountainous Soconusco region of Chiapas from January to April of 2014 (15°7.90 N, 92°16.46 W). This region of Chiapas is one of the largest coffee-growing regions in Mexico, with over 72,000 ha of land and millions of people dedicated to producing coffee [22,27]. We surveyed mammalian fauna and characterized vegetation within 23 sites in our 8250 km^2^ study area: 6 sites in BF coffee farms, 6 sites in conventional shade coffee that did not meet BF criteria, and 6 sites in sun coffee. Additionally, we surveyed 5 sites in nearby forest remnants, however as almost all the forest in this area at mid-elevation (500–1500 m) has been converted to coffee [5], the fragments remaining are small, isolated, highly compromised, and often limited to broken terrain inaccessible for growing coffee [9]. We selected sites that were homogeneous with these four habitat types and were not in close proximity to roadways, water, or residential areas to the best extent possible. The elevations of the sites ranged from 490 to 1070 m (mean 745 ± 31 m), with the coffee sites ranging from 640 to 1070 m (mean 790 ± 27 m). The sites were a minimum of 360 m apart from one another (mean 500 ± 54 m) except for two of the forest sites that were 150 m apart.

Bird Friendly sites were characterized as coffee sites that met the requirements for and have obtained the BF shade coffee certification (i.e. organic and >40% shade canopy cover). These sites would be classified as “rustic or traditional polyculture coffee” [28]. Conventional shade coffee sites were those that met the requirement of over 40% shade within the coffee, but were not organic and lacked the vegetation composition and structure required by the BF certification and fell into the “commercial polyculture” classification [28]. Sun coffee sites had less than 15% shade cover and were not organic. It should be noted that our sun coffee study sites were not devoid of shade trees and according to Philpott et al. [28] may fall into the classification of “shade monoculture coffee”—i.e., a single tree species providing scant, spotty shade throughout the farm [28]. All studies were carried out on private lands with permission and collaboration of the landowner.

### Mammal survey

At each 0.25-ha site, we established a sampling grid containing Sherman traps (8 x 8 x 23 cm) for small mammals, with a pair of Sherman traps placed 9-m apart throughout the grid. This configuration yielded a trap grid containing 50 Sherman traps; although due to the mountainous terrain some of the trap-grids were rectangular rather than square and contained 48 traps. The difference in trapping effort was taken into account in the analysis. For the most of the trap stations, we placed both small traps on the ground. However when low lying tree branches or lianas were available, we secured one trap approximately 1 m above the ground.

Each site was sampled once for 10 consecutive nights, with two to six sites surveyed at the same time. We varied the pairing of the habitat types to block for any climatic variation. All sampling took place during the dry season within a 3-month period, therefore any temporal effect of the sampling is negligible. The sampling sequencing was as follows: Session 1 –three forest sites; Session 2 –three BF and three sun coffee sites; Session 3 –three BF and three conventional shade coffee sites; Session 4 –three sun and three conventional shade coffee sites; Session 5 –two forest sites. Our effort yielded 11,220 trap nights during the dry season.

We baited the traps with a mix of peanut butter, bananas, vanilla, oats, seeds, and dry dog food and checked the traps daily, cleaned them of ants, and added bait as needed. We released all individuals trapped at the point of capture after determining the species, taking standard measurements such as length of body, tail, ear, hind foot, and weight [29] and ear tagging each individual with a unique identification number. We followed Reid [30] for field identifications and took mouth swabs and hair samples of individuals for DNA to genetically verify field identifications. The study did not involve endangered or protected species. All methods were approved by the Smithsonian Institution Animal Care and Use Committee and sampling procedures were reviewed as part of obtaining permits for this research.

In addition to the Sherman traps, one infrared camera trap was placed approximately 10 m outside of each trap grid. Cameras operated for 24 hours for the 10-day sampling duration at each site, yielding 230 camera trap nights for the study. The infrared camera (Bushnell Trophy Camera) with a passive infrared monitor was activated by heat and motion with an infrared flash not visible by the human eye. Photographs were identified to the species level and their capture subjects included in the total species density and estimated Chao 2 richness analysis.

### Habitat characteristics

We quantified vegetation characteristics at each site to assess possible factors associated with mammalian fauna. We measured the percent canopy cover with a concave spherical densiometer at five locations through the site. We used two 40-meter line intercepts at each site to categorize the sub-canopy structure. At each decimeter, we recorded the presence of vegetation at three levels: mid-stratum defined as understory plants and shrubs ≥1 m tall; lower stratum defined as weeds, grasses, plants, and understory shrubs from 5 cm to <1 m tall; and ground cover defined as grasses and weeds <5 cm tall. We recorded basal area of shade trees using a 10 factor wedge prism [31]. We counted the number of trees within each site for all habitats and additionally within the coffee sites, noted the tree species and tree height. For coffee habitats, we measured coffee plant height of two of the coffee rows. We noted the geographic coordinates and elevation within each site.

### Data Analysis

We analyzed mammal data as three dependent variables: small mammal abundance (number of individuals captured), small mammal species density (number of species detected per sampling grid), and total mammal species density (including medium and large mammals detected with camera traps) per site. The sampling unit for the analysis was the entire trap grid per site. All recaptured individuals (identified by their tag number) were removed for the analysis. The distance between each site was great enough to prevent spatial autocorrelation (as is evidenced by the fact that no individual was captured in two separate sites). We assessed the mean differences of these dependent variables among the four habitat categories of forest, BF coffee, conventional shade coffee, and sun coffee through Analysis of Variance (ANOVA), using Fisher’s Protected Least Square Difference (FPLSD) for multiple comparisons between treatment levels [32]. We used SAS Statistical Software version 9.3 [33] for all statistical modeling.

We also created Poisson regression models separately for small mammal abundance, small mammal species density, and total mammal species density at each site to examine the associations of the independent habitat variables: % canopy cover, % mid-strata vegetation, % lower strata vegetation, % ground cover, tree basal area (m^2^/ha), tree density (number of trees/m^2^), and tree richness, tree height (m), and coffee height (m) for the coffee sites only, with the dependent variables. For all ANOVA and regression models, the dependent variable for species richness comparisons was species density which is defined as the number of species within a specified area because the sampling area for each site was standardized [34].

The vegetation variables measured were summarized (either averaged or summed) for each site. Over or under dispersion in the data was adjusted by scaling for deviance. The variance inflation factor for each independent variable and the Pearson’s correlation was examined for evidence of multicollinearity. Because the tree measurements (tree species richness, tree density, basal area, tree height, and % canopy cover) were all highly correlated, we modeled each one separately with the cover measurements (% mid-strata vegetation, % lower strata vegetation, % ground cover). Those independent variables shown not to be significant were removed one at a time by backwards elimination. An offset of trap nights (number of traps x sampling nights) was included in all models to normalize data by sampling effort to account for the slightly unequal numbers of traps in the sites. The AIC was examined in each of the final models and the one with the lowest value was selected. We analyzed the difference in mean vegetation variables with regards to habitat type using ANOVA after the variables were normal transformed to meet the assumptions of ANOVA. FPLSD was employed for multiple comparisons for those models that were significant.

We conducted an analysis of similarity to compare the species composition among the habitat types of forest, BF coffee, shade coffee, and sun coffee using EstimateS Version 9.1.0 [35]. Additionally, we used EstimateS to calculate an estimate of species richness using the Chao 2 Incidence-Based Richness Estimator with presence/absence of species detected per observation (incidence frequencies) for both the small mammal data alone and then including the medium and large data from the camera traps to compare species richness among habitat types. The Chao 2 estimator provides a mean and 95% confidence interval of predicted species and takes into account rare, unseen species [36,37].

## Results

There were a total of 388 small mammal captures for the study period, which corresponded to 164 individuals, and 172 images of medium and large mammals from the camera traps We detected seven species small mammals through the Sherman traps and 10 species of medium and large mammals through the camera traps, yielding a total 17 species of mammals, representing 11 families (Table 1).

**Table 1:** Mammal species detected in each habitat type by Sherman traps and camera traps in a 2014 mammal survey in forest and coffee habitats in Chiapas, Mexico.

	Forest	Bird Friendly shade coffee	Conventional shade coffee	Sun coffee
**Family—Canidae**				
Urocyon cinereoargenteus*		**X**	**X**	
**Family—Cervidae**				
Mazama americana*		**X**	**X**	**X**
Odocoileus virginianus*		**X**	**X**	**X**
**Family—Cricetidae**				
***Handleyomys alfaroi***	**X**	**X**		
***Oligoryzomys fulvescens***				**X**
***Peromyscus gymnotis***	**X**	**X**	**X**	**X**
***Reithrodontomys mexicanus***		**X**	**X**	**X**
**Family—Cuniculidae**				
Cuniculus paca*	**X**			
**Family—Dasypodidae**				
Dasypus novemcinctus*	**X**		**X**	**X**
**Family—Dasyproctidae**				
Dasyprocta punctata*	**X**	**X**	**X**	**X**
**Family—Didelphidae**				
Didelphis marsupialis*	**X**	**X**	**X**	**X**
***Marmosa mexicana***	**X**	**X**		**X**
**Family—Felidae**				
Puma yagouaroundi*			**X**	
**Family—Heteromyidae**				
***Heteromys desmarestianus goldmani***	**X**	**X**	**X**	**X**
**Family—Procyonidae**				
Nasua narica*	**X**		**X**	
Procyon lotor*		**X**		
**Family—Soricidae**				
***Crypotis parva***		**X**		

* Species detected by camera traps alone

For all sites combined, 37.8% of all small mammal individuals captured were *Peromyscus gymnotis* (naked-eared deer mouse), 28.0% were *Handleyomys alfaroi* (Alfaro’s rice rat), and 14.0% were *Reithrodontomys mexicanus* (Mexican harvest mouse). Bird Friendly habitats were dominated by *H*. *alfaroi*, which accounted for 52.1% of all small mammal individuals captured, while *P*. *gymnotis* dominated the conventional shade coffee and sun coffee habitats, representing 55.6 and 65.7% of all small mammal individuals captured, respectively. Species composition within forest habitats was more evenly spread, although the two dominant species mirrored that of the coffee with *P*. *gymnotis* at 37.9% and *H*. *alfaroi* at 27.6%. All species captured were native to this region of Mexico.

### Habitat comparisons for mammal diversity

Overall, BF coffee habitats had the highest species density and abundance of mammals, although not always statistically significantly higher than the other habitats. We found a total of 10 species of mammals in forest, shade coffee, and sun coffee and 12 species in Bird Friendly coffee habitats (Table 1). Four species were shared by all habitat types (*Heteromys desmarestianus* (forest spiny pocket mouse), *P*. *gymnotis*, *Dasyprocta punctata* (Central American agouti), and *Didelphis marsupialis* (common opossum)) and an additional three species were found in all the coffee habitats (*R*. *mexicanus*, *Mazama Americana* (red brocket), and *Odocoileus virginianus* (white-tailed deer)).

We found a significant difference in mean species density for small mammals among the habitat types (F_3,19_ = 4.17, p = 0.020), with BF habitats hosting a significantly higher small mammal species density than the forest, shade, and sun coffee habitats (Table 2). The Bray-Curtis similarity index of species composition for small mammals was 71% for sun and shade coffee, 52.9% for forest and BF coffee, 50% for forest and sun coffee, 44% for BF and sun, 42.9% for forest and shade coffee, and 40% for BF and shade. There was no significant difference in the estimated species richness (Chao 2) among the different habitat types. For all mammals, the mean Chao 2 richness estimator was highest for shade coffee, followed by BF, sun, then forest, but each habitat type had large confidence intervals and none were significantly different than one another.

**Table 2:** Average values of mammal and vegetation measurements (± SE) within the four habitat categories surveyed in 2014 mammal study in Chiapas, Mexico.

	Forest	Bird Friendly shade coffee	Conventional shade coffee	Sun coffee
	(n = 5)	(n = 6)	(n = 6)	(n = 6)
**Small mammal abundance (# of individuals)**	5.8 ± 2.7	12.2 ± 2.7	4.5 ± 1.4	5.8 ± 2.7
**Small mammal species richness**	1.8 ± 0.4a*	3.3 ± 0.6b	1.5 ± 0.2a	1.8 ± 0.5a
**Medium-large mammal species density**	2.0 ± 0.3	2.7 ± 0.6	2.2 ± 0.7	1.5 ± 0.7
**Total mammal species richness**	3.8 ± 0.4a,b	6.0 ± 0.9a	3.7 ± 0.7b	3.3 ± 0.8b
**Canopy cover (%)**	93.3 ± 1.5a	86.0 ± 2.7a	49.9 ± 4.6b	9.9 ± 1.5b
**Basal area (m^2^/ha)**	25.5 ± 4.2a	26.9 ± 2.9a	21.6 ± 6.0a	6.2 ± 1.5b
**Mid-strata vegetation (%)**	50.4 ± 9.1	53.2 ± 5.9	46.8 ± 2.6	51.4 ± 3.2
**Lower strata vegetation (%)**	47.4 ± 5.8a	55.1 ± 10.2a	8.7 ± 2.4b	11.4 ± 4.8b
**Ground cover (%)**	17.6 ± 3.6	32.4 ± 12.7	49.0 ± 10.9	26.7 ± 6.6
**Tree density (#/site)**	157.3 ± 26.2a	62.0 ± 10.1b	40.4 ± 4.5c	24.8 ± 2.5c
**Tree species richness**	NA	9.7 ± 1.1	7.2 ± 1.3	5.8 ± 0.5
**Tree height (m)**	NA	16.3 ± 1.5a	15.3 ± 3.0a	5.9 ± 1.0b
**Coffee height (m)**	NA	2.6 ± 0.1a	2.1 ± 0.1b	2.0 ± 0.2b

* Letters (a, b, c) indicate significant difference (p≤0.05) among average measurements between habitat categories (same letter indicates no difference); n = number of sites for each habitat type.

For total mammal species density, both small and medium/large mammals, we found a significant difference among habitat types (F_3,19_ = 3.70, p = 0.030). BF sites had a higher mean species density than both shade and sun coffee sites but did not differ significantly from the forest sites (Table 2).

Bird Friendly habitat ranked the highest overall in relative abundance of small mammals, followed by forest, then sun coffee, and lastly shade coffee with 73, 28, 35, and 24 individuals per habitat type, respectively. However there was no significant difference in the mean abundance among the four habitat types studied.

### Vegetation characteristics associated with mammal diversity

We used Poisson log-linear regression models to examine the relationship between measured vegetation characteristics and the observed small mammal abundance and species density within the sites. Both small mammal abundance and species density was found to increase in sites that had greater amounts of lower strata vegetation (abundance: χ^2^ = 8.73 p = 0.003; density: χ^2^ = 15.69, p<0.001). Increases in shade tree basal area (χ^2^ = 3.93, p = 0.048) and lower strata vegetation (χ^2^ = 11.77, p<0.001) were found to significantly increase total species density.

### Vegetation comparisons across habitat types

We assessed the difference between the vegetation parameters measured within forest, BF coffee, shade coffee, and sun coffee habitats. Understandably, there were significant differences in almost all of vegetation characteristics that we measured (shade canopy cover: F_3,19_ = 143.27, p<0.001; shade tree basal area: F_3,19_ = 8.08, p = 0.001; tree density: F_3,19_ = 15.43, p<0.001; tree height: F_2,15_ = 9.12, p = 0.003, lower vegetation strata: F_3,19_ = 14.10, p<0.001, coffee height: F_2,15_ = 6.23, p = 0.011), although not all habitats were significantly different from one another (Table 2). Interestingly, the only vegetation variables that were significantly different from one another for sun and shade coffee habitats were shade tree height and basal area. In a comparison of BF coffee to conventional shade coffee, we found significantly higher amounts of shade canopy cover, tree density, lower vegetation strata, and coffee height in the BF habitats. Shade tree richness, basal area, and tree height did not differ significantly between the conventional shade coffee and Bird Friendly® coffee sites in this study.

## Discussion

Coffee farms provide an important refuge for mammalian wildlife in this agricultural region of Chiapas. These coffee farms serve as a high quality matrix in a landscape where the few remaining forest fragments are small, isolated, and highly compromised. The coffee farms we studied hosted an array of mammal species, although the BF coffee sites had significantly higher species density than the sun and conventional shade coffee farms.

The BF coffee sites we studied did host higher species density and abundance of mammals as compared to the sun coffee and conventional shade coffee sites, although not significantly so for small mammal abundance. Our findings suggest that while the criteria outlined for avian fauna in the BF certification also benefit mammals, requirements specifically devised to enhance mammal habitat could be included. BF standards of canopy cover, tree species richness, and tree height did not have a direct influence on the mammalian communities in this study. Medium and large mammals in our study were present where there were larger, more mature shade trees within the coffee associated with but not required by the BF certification standards which aligns with the other shade tree certification standards. However, lower strata vegetation (from 5 cm to 1 m tall), the only vegetation parameter found to increase abundance and species density for small mammal, is not specified in the BF standards. It may be that the requirements for the other vegetation parameters in conjunction with the prohibition of chemical herbicides per the organic certification encourage the presence of the vegetation in this lower stratum for which the small mammals benefit.

Small mammals are often prey species and as such may use ground-level vegetation as shelter and to escape predation. This finding is consistent with that of other non-volant mammal studies within coffee habitats that analyzed individual vegetative habitat parameters [10,38]. In Kodagu, India, Caudill et al. [38] found that small mammal species richness and abundance increased with greater amount of herbaceous ground cover (<5 cm in height). In coffee habitats in Costa Rica, small mammal richness and abundance were higher in sites with greater amounts of lower strata vegetation (from 5 cm to 1 m tall) [10]. Small mammals play an important part in the ecosystem, acting as seed dispersers, insectivores, and a large prey base for predatory mammals, snakes, and birds.

To enhance the habitat for small non-volant mammals, a criterion pertaining specifically to the amount and structure of lower strata vegetation cover should be incorporated. This standard relating to ground cover has not been taken into account when devising standards for avian habitat, but could obviously be pertinent to ground-foraging birds. It should be noted that creating vegetation cover for small mammals might also create cover for other taxa of safety concerns to the farm workers, such as poisonous snakes, a commonplace group in many coffee-growing regions. It is therefore important to consider the human element in these agricultural landscapes when devising sustainable standards to support biodiversity.

There were not clear lines of the effect of management intensity on the mammalian fauna in our study. We found no significant difference in terms of small mammal abundance among the four habitat types. Mammal species density was not significantly different among forest, sun, or conventional shade coffee, although it was significantly higher in Bird Friendly coffee. Similarly, Gallina et al. [12] found no detectable relationship for the small mammals with regards to management intensity within coffee farms, but species composition of medium-sized mammals varied inversely with intensification. The authors recommended maintaining diverse shade tree richness and structure within coffee farms, as well as reducing the use of pesticides.

The BF and forest sites in our study were agrochemical-free, whereas the sun and conventional shade used agrochemicals. Because so many of the vegetation variables that we measured among the sites were significantly different, it would be difficult to ascertain the effect of the chemical inputs on the mammal community. A future study to assess the effects of agrochemicals on non-volant mammals, all other habitat characteristics being similar, is an obvious research need.

Although our study was devised to measure farm-level characteristics associated with mammal diversity, it is important to note that broader landscape characteristics play a role in mammal diversity within agroforestry systems [10,39]. In contrast to volant taxa such as birds that can move easily from one habitat patch to another, mammals are susceptible to extinction when patch size falls below certain species-specific thresholds. Small non-volant mammals, in particular, have limited home ranges and dispersal mobility, therefore resources for their survival must be within these home ranges and must be sufficiently connected at landscape scales to permit species movement/migration. Studies have shown that the number of mammal species increases in agroforestry systems as the distance to native forests decreases for coffee study sites in India [40] and also in cocoa agroforestry in Indonesia [41]. Additionally, Caudill et al. [10] found that mammal density and richness decreased with increased proportions of sun coffee within the landscape and increased as the amount of shade coffee increased in Costa Rica. Furthermore, the small mammals thrived in areas adjacent to forest patches and as the proportion of forested areas within the landscape increased. Preserving or reestablishing forested areas embedded within coffee landscapes is imperative to enhance or maintain mammal diversity. However, we note that establishing and monitoring this criterion within farm-level certifications, such as Bird Friendly, presents a challenge. To protect taxa that are dependent on forest areas and connectivity across landscapes, we may need to expand the scale of our conservation efforts and understand how certified farms can fit into a suite of conservation tools on a landscape level.

Our study was preliminary in nature and further research is clearly needed to understand the dynamics of mammal communities in coffee landscapes. We certainly concede that detecting rare species of mammals in tropical landscapes is difficult and it is likely that rare species are not well represented in our data. Future studies could increase the likelihood of detecting rare species by increasing the sampling duration and effort per site. Furthermore, a more robust sampling effort would be recommended for sampling mammals for certification evaluation, particularly for camera traps. We would also recommend incorporating a sampling design that includes a landscape perspective in addition to individual coffee farms for future certification studies [42].While the number of non-volant mammal studies has been increasing within coffee agroforestry, we are just beginning to understand the response of mammal communities to these changing habitats within coffee landscapes. Our study indicates that in fragmented landscapes with few isolated forested areas remaining, mammals are taking refuge and are thriving within coffee farms. Overall management strategies to protect avian habitat within coffee farms seem to protect mammal habitat as well, although additional standards and an approach directed at a landscape scale that accounts for larger habitat patches should be considered to enhance important habitat features for the non-volant mammals that rely on this important refuge within agricultural landscapes.
